# Disparities in cancer survival by socioeconomic status: findings from a population-based study of 942 241 Australians from 1980 to 2019

**DOI:** 10.1093/jnci/djaf305

**Published:** 2025-11-13

**Authors:** Sarsha Yap, Qingwei Luo, Jeff Cuff, David Goldsbury, Xue Qin Yu, Yoon-Jung Kang, Benjamin D T Gallagher, Eleonora Feletto, Marianne Weber, Preston Ngo, Melissa A Merritt, Karen Canfell, David P Smith, Julia Steinberg

**Affiliations:** The Daffodil Centre, The University of Sydney, and Cancer Council NSW, Sydney, New South Wales, Australia; The Daffodil Centre, The University of Sydney, and Cancer Council NSW, Sydney, New South Wales, Australia; Faculty of Science Biotech and Biomolecular Science, University of New South Wales, Sydney, New South Wales, Australia; Research advocate, The Daffodil Centre, The University of Sydney, and Cancer Council NSW, Sydney, New South Wales, Australia; The Daffodil Centre, The University of Sydney, and Cancer Council NSW, Sydney, New South Wales, Australia; The Daffodil Centre, The University of Sydney, and Cancer Council NSW, Sydney, New South Wales, Australia; The Daffodil Centre, The University of Sydney, and Cancer Council NSW, Sydney, New South Wales, Australia; The Daffodil Centre, The University of Sydney, and Cancer Council NSW, Sydney, New South Wales, Australia; The Daffodil Centre, The University of Sydney, and Cancer Council NSW, Sydney, New South Wales, Australia; The Daffodil Centre, The University of Sydney, and Cancer Council NSW, Sydney, New South Wales, Australia; Cancer Surveillance Branch, International Agency for Research on Cancer, Lyon, France; The Daffodil Centre, The University of Sydney, and Cancer Council NSW, Sydney, New South Wales, Australia; School of Public Health, The University of Sydney, Sydney, New South Wales, Australia; The Daffodil Centre, The University of Sydney, and Cancer Council NSW, Sydney, New South Wales, Australia; The Daffodil Centre, The University of Sydney, and Cancer Council NSW, Sydney, New South Wales, Australia

## Abstract

Cancer survival in Australia has improved over time, but disparities by socioeconomic status persist. We analyzed data from the population-wide New South Wales Cancer Registry, including 942 241 individuals with invasive solid cancers diagnosed between 1980 and 2019. We examined cancer-specific and all-cause deaths by area-based socioeconomic status for all solid cancers and 12 common cancers using competing risks regression alongside crude survival. Five-year cancer-specific survival for all cancers improved from 50.3% in 1980-1989 to 73.3% in 2010-2019. Risk of cancer death was higher for individuals living in most socioeconomically disadvantaged areas (vs least disadvantaged areas), and differences increased over time: from a sub-hazard ratio of 1.04 (95% CI = 1.02 to 1.07) in 1980-1989 to 1.35 (95% CI = 1.32 to 1.38) in 2010-2019 (adjusting for age, sex, cancer type, cancer spread). Statistically significant and increasing differences were observed for prostate, breast, melanoma, colorectal, lung, bladder, and stomach cancers. Disparities in cancer survival have continued to widen, requiring improved understanding and targeted interventions to address inequities.

Cancer survival has increased in many countries over recent decades^1^^,^^2^; in Australia, 5-year relative survival for all cancers increased from 51% in 1988-1992 to 70% in 2013-2017.^3^ Despite these gains, improvements have not been experienced equally across all population groups. Australian studies have documented disparities in cancer survival by socioeconomic status (SES) for all cancers combined^4-7^ and for specific cancers.^4^^,^^6^^,^^8-14^ A study of New South Wales data for 1980 to 2008^4^ found that among individuals diagnosed with cancer, persons living in the most socioeconomically disadvantaged areas had a higher risk of cancer-specific death than persons in the least disadvantaged areas. Notably, this study reported a widening gap over time. With equity in outcomes being a key national policy goal,^15^ it is vital to examine the most recent data to determine the latest trends.

Building on past work,^4^ we extended the analysis up to 2019 and examined 40 years of cancer survival trends by SES. We used the statewide New South Wales Cancer Registry (NSWCR), which covers the most populous Australian state (one-third of the national population), with age-standardized cancer incidence similar to Australia.^16^ We completed comprehensive survival analyses of cancer-specific deaths as the primary outcome, analyzing all solid cancers combined and each of the 12 cancer types with the highest numbers of new diagnoses in Australia in 2023 (“most common cancers”).^17^ As a secondary outcome, we completed analyses of all-cause deaths.

Specifically, we analyzed survival of all individuals diagnosed with cancer in New South Wales between 1980 and 2019, using data from the Cancer Institute NSW Enduring Cancer Data Linkage (CanDLe).^18^ CanDLe includes NSWCR records (1972-2021) linked to additional death (1985-2021) and hospital records (2001-2019).^18^ This study was approved by the CanDLe Community of Practice and the Population Health and Services Research Ethics Committee (approval CCNSW001/005, project 2019/ETH12584). Supplementary Methods detail all data sources and methods.

Briefly, individuals diagnosed with invasive solid cancers and each of the 12 most common cancers were identified using *International Statistical Classification of Diseases and Related Health Problems, Tenth Revision, Australian Modification* codes (Table S1; for full exclusion criteria, see Figure S1). Following a previous approach,^4^ we excluded individuals diagnosed with brain and central nervous system cancers (codes C70-72) and lymphohematopoietic cancers (codes C81-96), for which spread of disease is generally unavailable in the data, as well as individuals with cancers identified by death certificate only.

Cancer-specific survival was defined as time from first cancer diagnosis to death from cancer; overall survival was defined as time to death from any cause. For analysis of a specific cancer type, cancer-specific survival was the time from first diagnosis of that cancer to death from that specific cancer, with deaths due to other causes (including other cancers) treated as competing events. Persons who were alive at the end of follow-up (ie, December 15, 2021) were censored at that time. For analysis of a specific cancer type, individuals with multiple diagnosis records for different cancers were included in each relevant analysis (see Supplementary Methods). For analysis of all solid cancers combined, individuals were included based on their first primary cancer diagnosis only.

Socioeconomic status at cancer diagnosis was based on the Census-derived area-based Index of Relative Socio-Economic Disadvantage^19^ composed of 15 indicators; lower scores indicate higher proportions of relatively disadvantaged people (eg, lower household incomes, low-skilled occupations).^20^ Socioeconomic status was categorized into quintiles from 1 (most disadvantaged) to 5 (least disadvantaged).^4^^,^^21^^,^^22^ For each individual, we also considered year of cancer diagnosis, age, sex, spread of disease, and area-based remoteness of residence,^23^ all at the time of diagnosis. The Charlson Comorbidity Index (excluding cancer diagnoses) was derived using hospital data^24^^,^^25^; for a sensitivity analysis, we also derived an adapted version of the National Comorbidity Index using the same data^26^^,^^27^(Supplementary Methods, Table S2). Multiple imputation was used as previously described^28^ to impute spread of disease where unknown. For all solid cancers combined, 16.4% of individuals had “unknown” spread of disease at diagnosis. Among the 12 most common cancers, this proportion ranged from 4.5% for melanoma to 34.2% for prostate cancer (see Table S3, Supplementary Methods).

We estimated 1-, 2-, and 5-year crude survival (cancer specific and overall) by diagnostic decade, calculated as 1 minus the cumulative incidence function.^29^

Associations between deaths and SES were assessed using competing risks regressions, estimating sub–hazard ratios (SHRs) and 95% CIs. This approach models cancer-specific deaths while accounting for competing noncancer deaths and allows simultaneous adjustment for several key covariates considered in this study (age, sex, remoteness of residence, comorbidities); in our study, this approach uses high-quality cause-of-death information in the NSWCR.^30-32^ Regression models were stratified by diagnostic decade, adjusted for SES, sex, age at diagnosis, and cancer type (“minimally adjusted”); additional models adjusted for cancer spread (after multiple imputation). To verify trends over time, separate nonstratified analyses tested for interactions between diagnosis year (as a continuous variable) and SES, using the Wald ꭓ^2^ tests (Supplementary Methods).

Sensitivity analyses were undertaken to verify the robustness of results, including additional adjustments for remoteness of residence and/or comorbidities (Charlson Comorbidity Index or National Comorbidity Index), and analyses of selected cancers excluding localized disease (Supplementary Methods).

Analyses were conducted using SAS, version 9.4, software (SAS Institute Inc); R, version 4.3.2, software (R Foundation for Statistical Computing); and Stata, version 18, software (StataCorp).

After exclusions, we included 942 241 individuals (Figure S1), with a median age at diagnosis of 66 years (IQR, 56-75 years). At diagnosis, 44.2% of individuals had localized disease (53.5% after multiple imputation), 54.2% were male, and 22.3% lived in the most disadvantaged areas (vs 18.0% in the least disadvantaged) (Table S3). As of December 15, 2021, 42.9% of individuals had died from cancer, 17.7% had died from other causes, and 39.5% were alive. Of individuals living in the most disadvantaged areas, 25.1% and 37.2% died from cancer within 1 and 5 years after diagnosis, respectively (vs 14.4% and 25.2% in the least disadvantaged areas, respectively). The number of individuals diagnosed with specific cancers ranged from 13 146 for liver cancer to 167 013 for prostate cancer (Table S3).

From 1980-1989 to 2010-2019, cancer-specific survival increased for all solid cancers combined and for 11 of the 12 most common cancers (except bladder) (Figures S2 and S3, Supplementary Results and Discussion). As survival estimates can be affected by changes in cancer type distribution,^33-35^ we also constructed weighted estimates, with highly similar results (Supplementary Methods, Supplementary Results and Discussion). For all solid cancers combined, crude 5-year cancer-specific survival improved from 50.3% in 1980-1989 to 73.3% in 2010-2019 (Figure S2a, Table S4; consistent with national estimates^3^). Prostate, colorectal, kidney, liver, and stomach cancers showed the largest increases in 5-year cancer-specific survival. Overall survival showed similar trends to cancer-specific survival (Figures S2 and S4, Table S4).

For all solid cancers combined, risk of cancer death increased with area-based disadvantage (Figure 1, A; Table S5). Disparities by SES differed over time (*P*_interaction_ < .001) and were substantially higher in 2010-2019 than in 2000-2009 (nonoverlapping 95% CIs)—for example, most vs least disadvantaged areas SHR = 1.42 (95% CI = 1.39 to 1.45) vs SHR = 1.25 (95% CI = 1.22 to 1.27) in minimally adjusted models, with analogous increases in association for SES quintiles 2 and 3 vs 5 (least disadvantaged) over that period. Estimates diminished only slightly when additionally adjusted for cancer spread at diagnosis (eg, most vs least disadvantaged areas, 2010-2019: SHR = 1.35, 95% CI = 1.32 to 1.38) (Figure 1, B) and with further adjustment for remoteness and comorbidities (Charlson Comorbidity Index—eg, most vs least disadvantaged areas, 2010-2019: SHR = 1.31, 95% CI = 1.28 to 1.34) (for full results, see Supplementary Results and Discussion). Generally, sensitivity analyses indicated that although additional adjustments led to some attenuations in subhazard ratios for SES, SES-associated differences in cancer-specific survival remained statistically significant (95% CIs not including 1) (Supplementary Results and Discussion). Similar patterns were observed for risk of all-cause death (Figures S5 and S6, Table S6).

**Figure 1.**
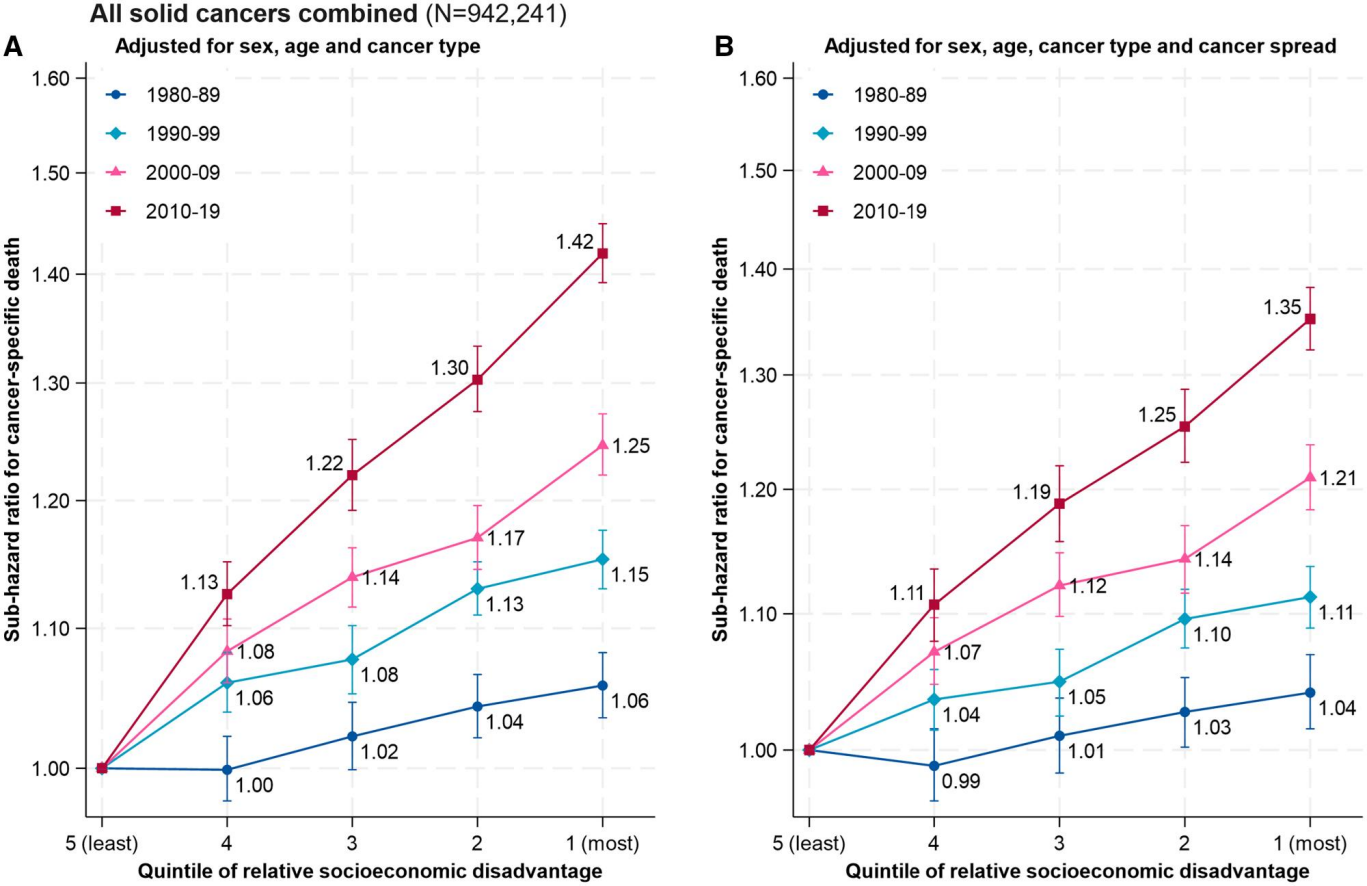
Association between socioeconomic disadvantage and cancer-specific deaths for all solid cancers combined, by 10-year period of diagnosis. **(A)** Association results for cancer-specific deaths, adjusted for sex, age, and cancer type. **(B)** Association results for cancer-specific deaths, adjusted for sex, age, cancer type, and cancer spread at diagnosis. Estimates show multivariable adjusted subhazard ratios, with bars showing 95% CIs. Subhazard ratios are shown on a log scale (y-axis). Detailed estimates are provided in Table S5.

In 2010-2019, risk of cancer death was also statistically significantly higher with greater area-based disadvantage for 10 of the 12 most common cancers (prostate, breast, melanoma, colorectal, lung, pancreatic, uterine, bladder, liver, and stomach cancers) (Figure 2; for further details, see Supplementary Results and Discussion, Table S5). For 7 of these cancers (except pancreatic, uterine, and liver cancer), disparities by SES widened between 2000-2009 and 2010-2019 (Supplementary Results and Discussion), with variations in effect across time supported by statistically significant interactions between diagnosis year and SES (*P* ≤ .001). In 2010-2019, survival differences for the most vs least disadvantaged areas were largest for prostate cancer, with SHR = 1.58 (95% CI = 1.44 to 1.74), increased from SHR = 1.29 (95% CI = 1.20 to 1.39) in 2000-2009 (additionally adjusted for cancer spread) (Figure 2, Table S5). Notably, the strong association for 2010-2019 remained after excluding individuals with localized disease at diagnosis (most vs least disadvantaged areas: SHR = 1.52, 95% CI = 1.33 to 1.72) (Table S7). Similarly, for breast cancer and melanoma, survival disparities for most vs least disadvantaged areas in 2010-2019 were slightly reduced but remained statistically significant when excluding individuals with localized disease at diagnosis (Supplementary Results and Discussion). Patterns of survival disparities by SES were similar for all-cause deaths (Figure S5, Table S6). Results of all sensitivity analyses were consistent with the main analyses (Supplementary Results and Discussion, Table S7).

**Figure 2.**
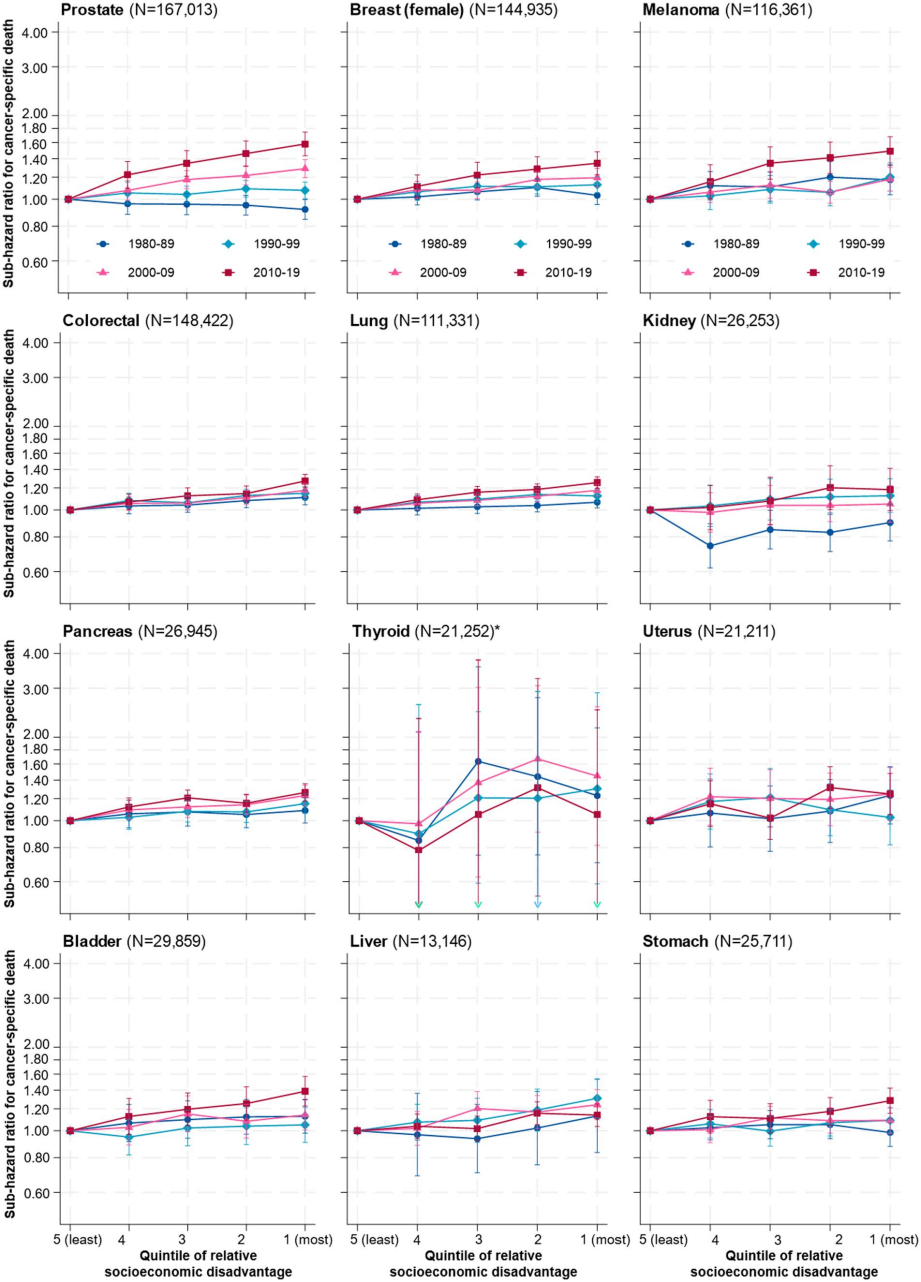
Association between socioeconomic disadvantage and cancer-specific deaths for each of the 12 most common cancers, by 10-year period of diagnosis. The figure shows adjusted subhazard ratios, with bars showing 95% CIs. Estimates for prostate, breast (female), and uterine cancers were adjusted for age at diagnosis and cancer spread at diagnosis; all other estimates were adjusted for sex, age at diagnosis, and cancer spread at diagnosis. Subhazard ratios are shown on a log scale (y-axis). ^a^CIs indicated by arrows extend beyond the y-axis limits. Detailed estimates are provided in Table S5.

Overall, our results show that although cancer survival has improved over a 4-decade period, disparities in cancer-specific survival by SES have continued to widen since previous reports,^4^ for all solid cancers combined and for many different cancer types. Previous reviews attribute SES-associated survival differences to cancer stage at diagnosis, comorbidities, and remoteness.^7^^,^^36^ In this study, adjusting for these factors attenuated but did not eliminate disparities (Supplementary Results and Discussion), suggesting a multifactorial basis that possibly included differences in overall health, treatment, additional tumor characteristics, and other factors.^4^^,^^6^^,^^7^^,^^9^^,^^36-39^ Our findings align with Australian^4^^,^^8^ and international studies^7^^,^^36^^,^^38^^,^^40^^,^^41^ that indicate widening SES differences in survival.

Although prostate cancer showed the highest absolute increases in crude survival, differences by SES were particularly pronounced; such survival differences were previously linked to differences in prostate-specific antigen testing behaviors (testing more common for higher SES^42^), stage at diagnosis, and treatment.^43^^,^^44^ Different early detection behaviors resulting in different lead time bias (earlier detection without delaying death) and overdiagnosis could also contribute to survival differences by SES.^44^^,^^45^ Consistent with these aspects, for prostate cancer, breast cancer, and melanoma, excluding individuals with localized disease at diagnosis attenuated associations between SES and survival (particularly before 2010); nonetheless, strong associations for 2010-2019 remained. Future research using detailed histopathological data to assess disease aggressiveness may provide greater insight into the relationships among SES, overdiagnosis, and survival.

This study has limitations. Crude survival was estimated using data to December 15, 2021; thus, estimates of 5-year survival for cases diagnosed between 2010 and 2019 may change once the full 5-year follow-up data (ie, to December 2024) for all individuals are available. The study is descriptive, with association estimates not to be interpreted as causal. The subhazard ratio estimates from different models are not directly comparable, and use of multiple imputation has some limitations (Supplementary Results and Discussion). Data on clinical trial enrolment were unavailable; differential access by SES might contribute to survival differences. Geographical areas change over time,^21^^,^^22^ and we used an area-based measure of relative SES determined by diagnosis year and relevant Census year; however, our analysis used wider SES groupings (Index of Relative Socio-Economic Disadvantage quintiles, not specific scores), which are more stable across time. In addition, information about Aboriginal and Torres Strait Islander (Indigenous) status was not available in our study, so we could not assess outcomes separately for Indigenous people or quantify the overlap between Indigenous status and SES in New South Wales. Aboriginal and Torres Strait Islander people make up approximately 3.4% of the New South Wales population,^46^ are more likely to live in disadvantaged areas,^47^ and have poorer cancer outcomes than non–Indigenous Australians^3^; this overlap may contribute to the disparities observed here but is unlikely to fully explain them. Incomplete 5-year follow-up for diagnoses from 2017 may affect survival estimates for 2010-2019. New South Wales data may not be entirely representative of Australia, though crude survival estimates were similar.^3^

This study also has notable strengths. We analyzed statewide linked data covering 4 decades to provide comprehensive insights into cancer survival by SES. Competing risks regression is particularly important when including older individuals who have a higher risk of noncancer death, especially if that risk also varies by SES.^4^

In conclusion, this study provides benchmarks of cancer survival by SES to measure future progress in cancer control. Our findings emphasize the need for targeted actions to understand and address widening SES differences in cancer survival.

## Supplementary Material

djaf305_Supplementary_Data

## Data Availability

The CanDLe-1 cohort and linked datasets can be obtained from the relevant data custodians for approved research projects, and enquiries for data access can be made to the Cancer Institute NSW (see https://www.cancer.nsw.gov.au/research-and-data/cancer-data-and-statistics/data-available-on-request/candle-program/how-to-access-the-candle-data/candle-data for details). These are third-party data not owned or collected by the authors and cannot be passed on by the authors themselves.
