# Supplementary material for: Disparities in cancer survival by socioeconomic status: findings from a population-based study of 942 241 Australians from 1980 to 2019
Source: J Natl Cancer Inst. 2025 Nov 13;118(7):1313–9. doi: 10.1093/jnci/djaf305 (PMC13339099; doi:10.1093/jnci/djaf305)
Supplement: djaf305_Supplementary_Data [file djaf305_supplementary_data.zip › CancerSurvivalSES_SupplementaryMaterials_Cleaned.pdf]

Disparities in cancer survival by socioeconomic status: findings from a population-based study of 942 241 Australians from 1980 to 2019

Sarsha Yap[1], MBiostat, Qingwei Luo[1], PhD, Jeff Cuff[2,3], David Goldsbury[1], MPH, Xue Qin Yu[1], PhD, Yoon-Jung Kang[1,6], PhD, Benjamin D.T. Gallagher[1], PhD, Eleonora Feletto[1,6], PhD, Marianne Weber[1,6], PhD, Preston Ngo[4], PhD, Melissa A. Merritt[1], PhD, Karen Canfell[5], DPhil, David P. Smith[1], PhD, Julia Steinberg[1*], DPhil

1 The Daffodil Centre, The University of Sydney, and Cancer Council NSW, Sydney, New South Wales, Australia.
2 Faculty of Science Biotech and Biomolecular Science, University of New South Wales, Sydney, New South Wales, Australia.
3 Research advocate, The Daffodil Centre, The University of Sydney, and Cancer Council NSW, Sydney, New South Wales, Australia.
4 Cancer Surveillance Branch, International Agency for Research on Cancer, Lyon, France.
5 School of Public Health, the University of Sydney, Sydney, New South Wales, Australia.
6 Current address: School of Public Health, the University of Sydney, Sydney, New South Wales, Australia.

Supplementary Materials

Supplementary Figures **2**

Figure S1. Study population: individuals diagnosed with invasive solid cancer between 1980 and 2019 in NSW, Australia. 2

Figure S2. Crude cancer-specific and overall survival for 942,241 individuals diagnosed with invasive solid cancer between 1980 and 2019 in NSW, Australia. 3

Figure S3. Crude 1-, 2- and 5-year cancer-specific survival for each of 12 most common cancers, by 10-year period of diagnosis. 4

Figure S4. Crude 1-, 2- and 5-year overall survival for each of the 12 most common cancers, by 10-year period of diagnosis. 5

Figure S5. Association between socioeconomic disadvantage and all-cause deaths for all solid cancers combined, by 10-year period of diagnosis 6

Figure S6. Associations between socioeconomic disadvantage and risk of all-cause death for each of the 12 cancer types, by 10-year period of diagnosis. 7

Supplementary Methods **8**

Supplementary Results and Discussion **15**

References **21**

Supplementary Figures

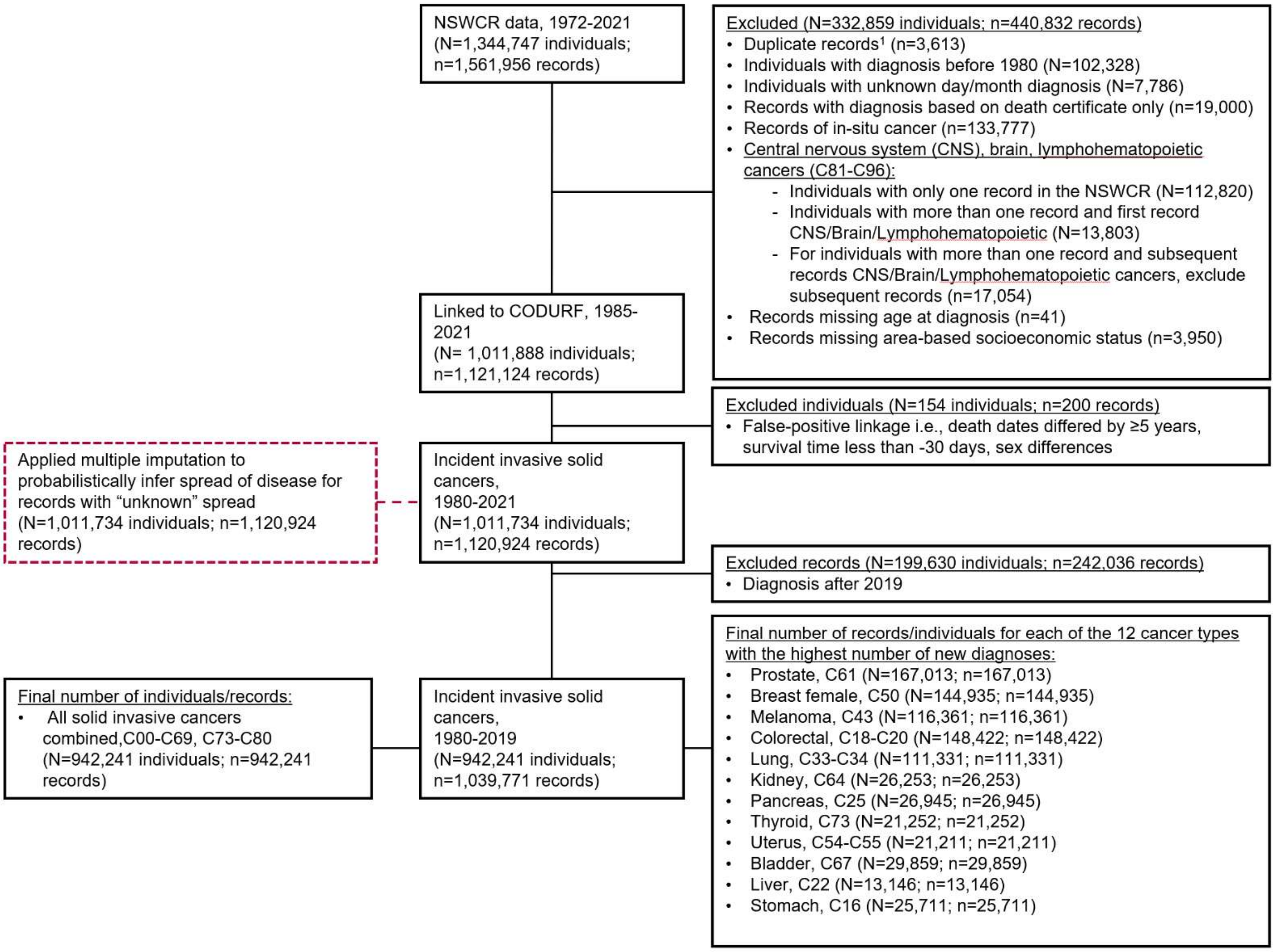

Figure S1. Study population: individuals diagnosed with invasive solid cancer between 1980 and 2019 in NSW, Australia.

Throughout, ‘N=’ and ‘n=’ denote numbers of individuals and records, respectively.

[1] Here, determined based on same individual ID (Project Person Number, “PPN”), cancer diagnosis date, cancer type, diagnosis age, sex, spread of disease, date of birth, death date, underlying cause of death, socioeconomic disadvantage quintile and remoteness of residence category

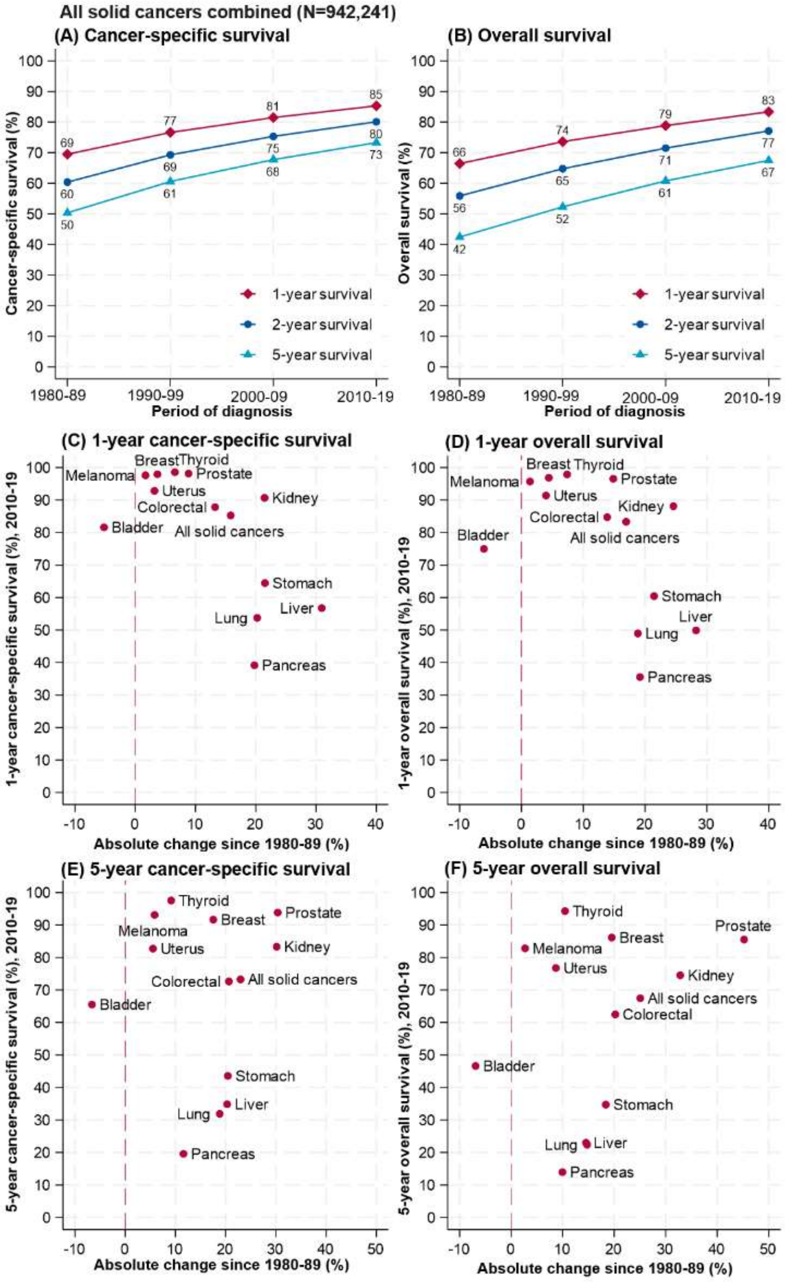

Figure S2. Crude cancer-specific and overall survival for 942,241 individuals diagnosed with invasive solid cancer between 1980 and 2019 in NSW, Australia.

(A) Cancer-specific survival by diagnostic decade

(B) Overall survival by diagnostic decade

(C) 1-year cancer-specific survival for those diagnosed 2010-19 and absolute change compared to survival for those diagnosed 1980-89

(D) 1-year overall survival for those diagnosed 2010-19 and absolute change compared to survival for those diagnosed 1980-89

(E) 5-year cancer-specific survival for those diagnosed 2010-19 and absolute change compared to survival for those diagnosed 1980-89

(F) 5-year overall survival for those diagnosed 2010-19 and absolute change compared to survival for those diagnosed 1980-89

The absolute change in survival was the absolute difference between survival estimates for those diagnosed in 2010-19 versus 1980-89. Survival estimates for each of the 12 most common cancers across time are shown in Figures S3-S4, with all detailed estimates provided in Table S4.

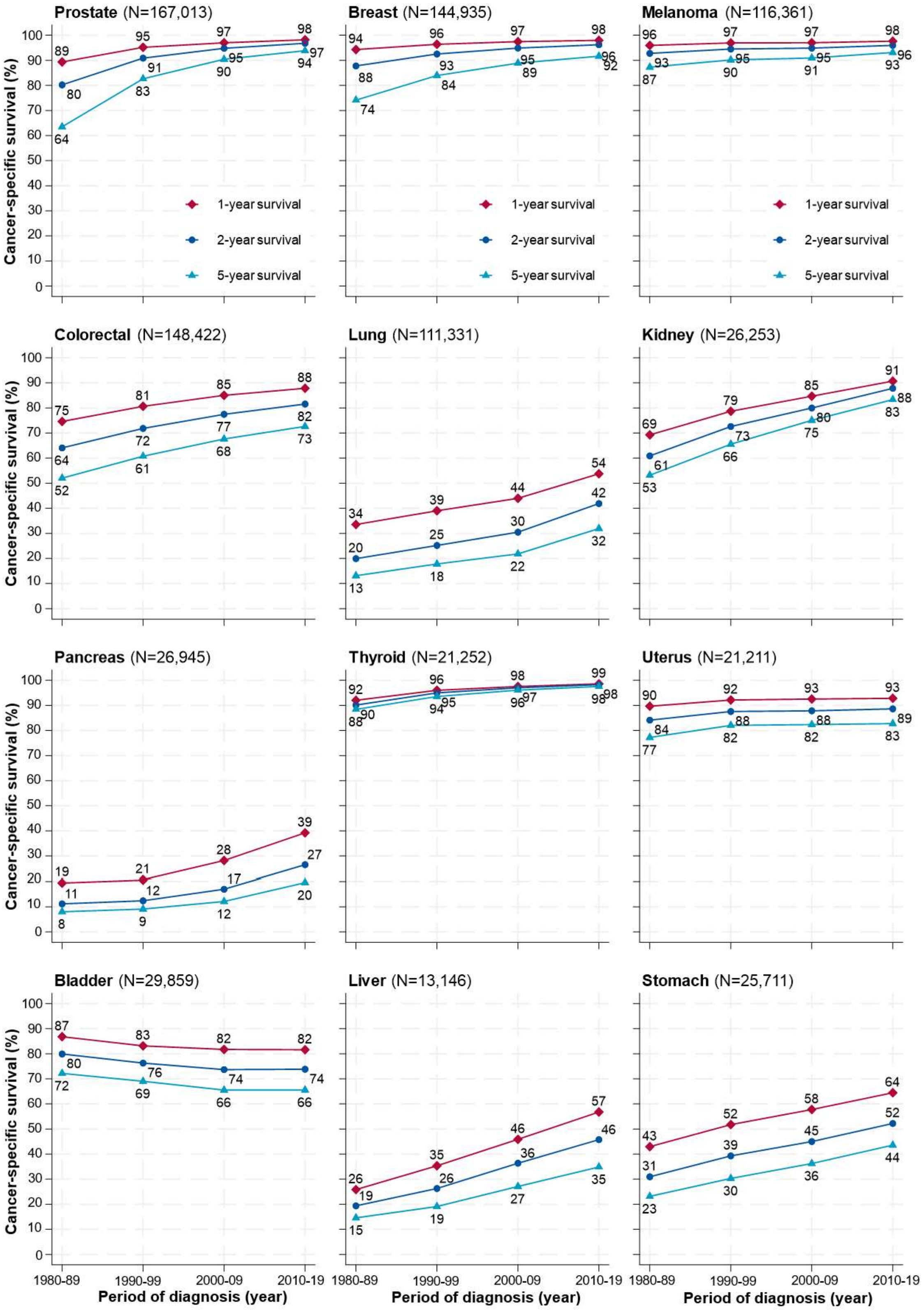

Figure S3. Crude 1-, 2- and 5-year cancer-specific survival for each of 12 most common cancers, by 10-year period of diagnosis. Crude survival was estimated using 1 minus the cumulative incidence function, accounting for competing risks of death.

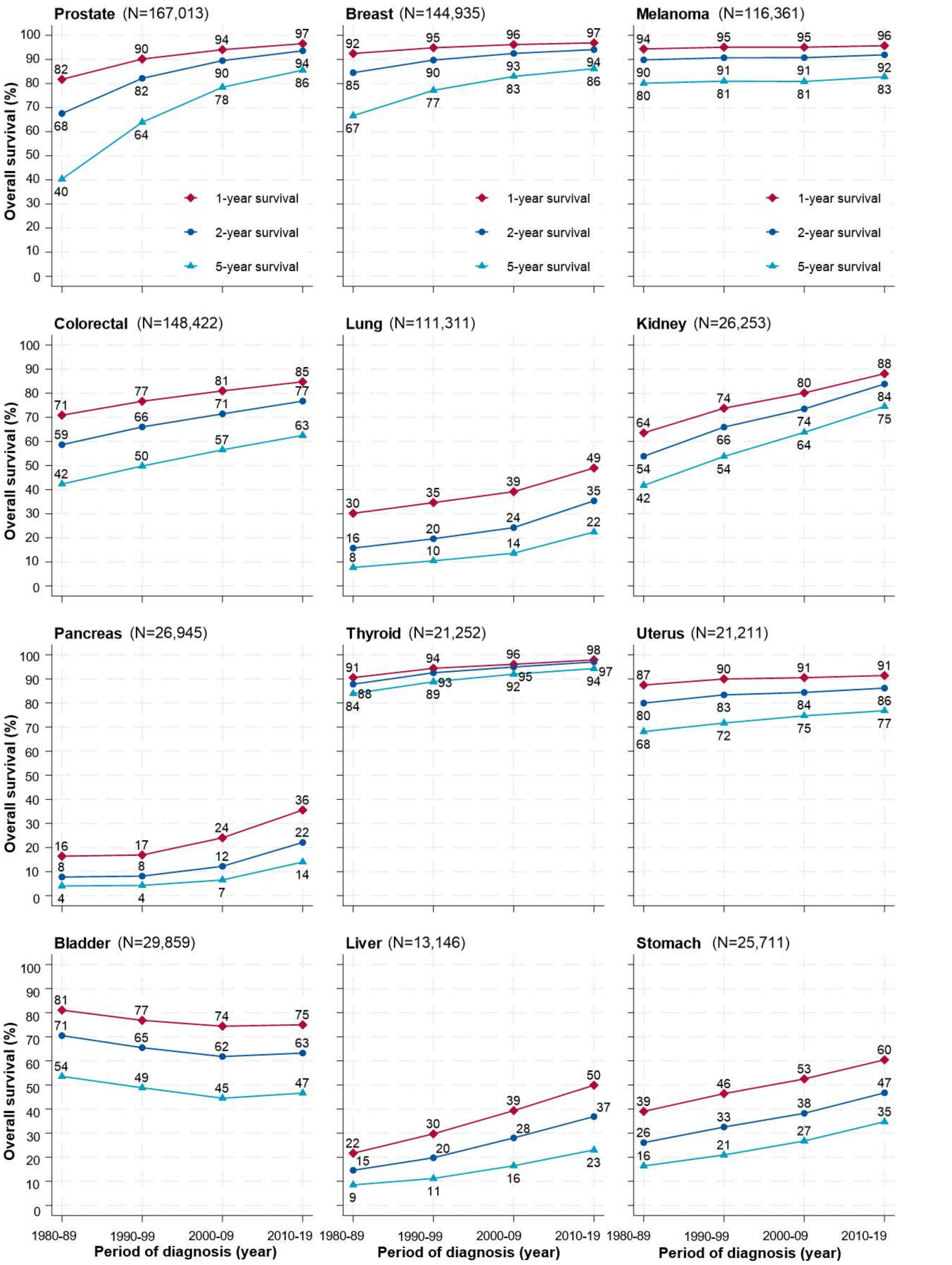

Figure S4. Crude 1-, 2- and 5-year overall survival for each of the 12 most common cancers, by 10-year period of diagnosis. Crude survival was estimated using 1 minus the cumulative incidence function.

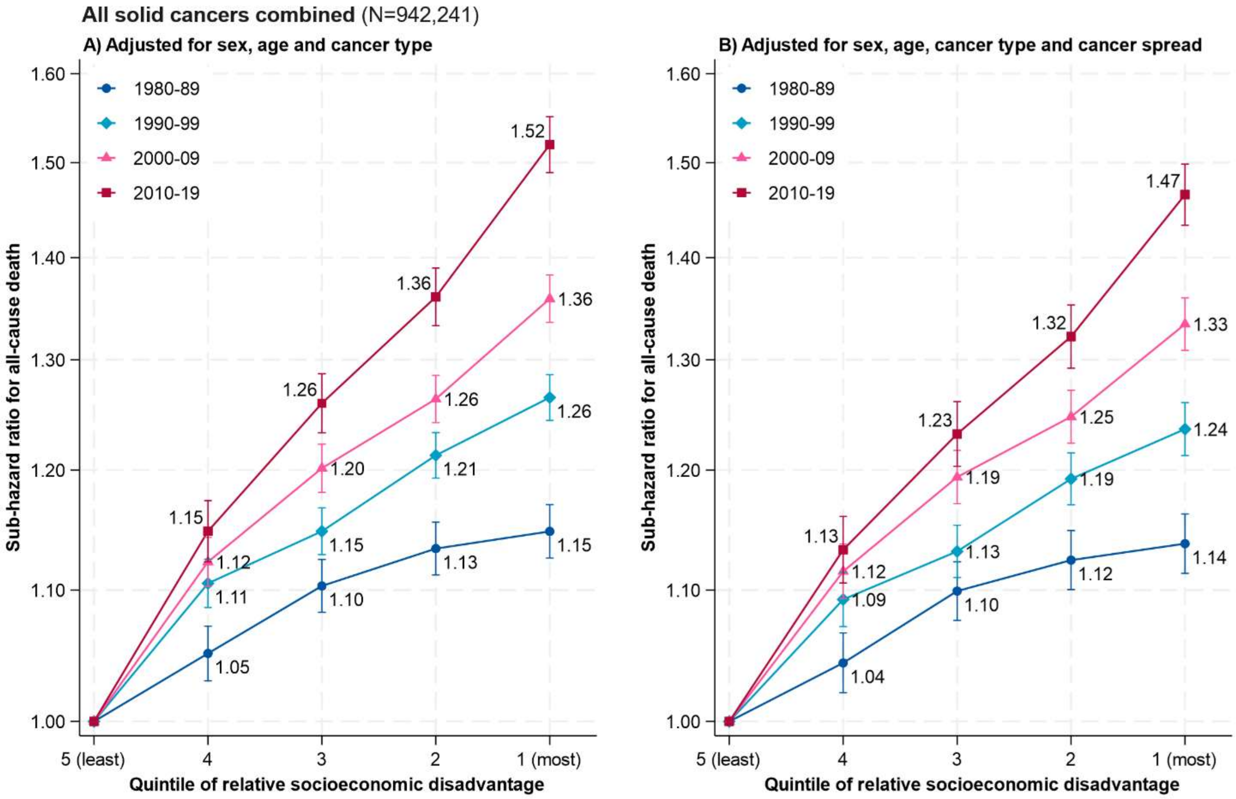

Figure S5. Association between socioeconomic disadvantage and all-cause deaths for all solid cancers combined, by 10-year period of diagnosis.

(A) Association results for all-cause deaths, adjusted for sex, age, and cancer type. **(B)** Association results for all-cause deaths, adjusted for sex, age, cancer type, and cancer spread at diagnosis. Estimates show multivariable adjusted sub-hazard ratios (SHRs), with bars showing 95% confidence intervals (CIs). SHRs are shown on a log scale (y axis). Detailed estimates are provided in Table S6.

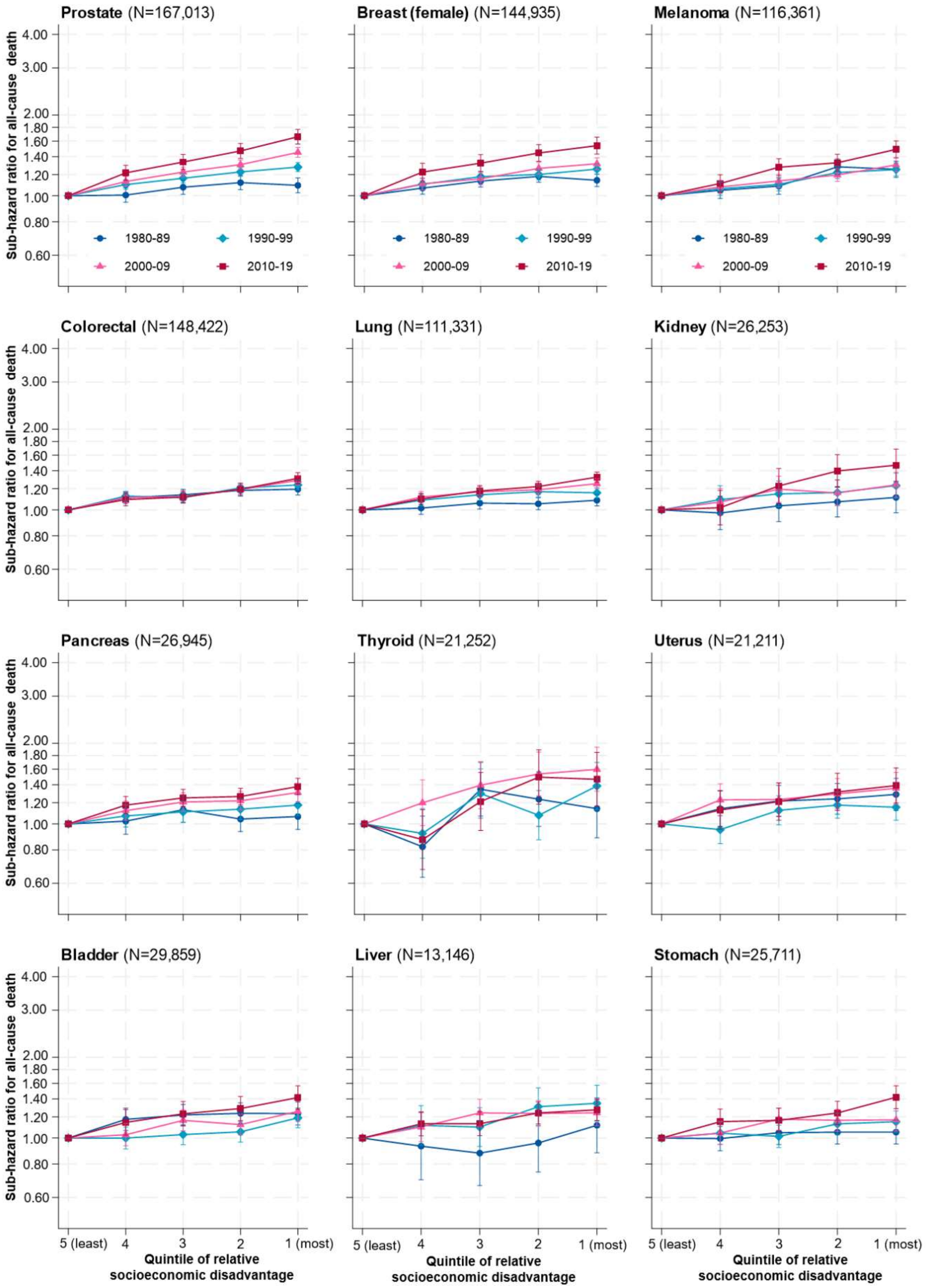

Figure S6. Associations between socioeconomic disadvantage and risk of all-cause death for each of the 12 cancer types, by 10-year period of diagnosis.

The figure shows adjusted sub-hazard ratios (SHRs) and 95% confidence intervals (shown by bars). Estimates for prostate, breast (female) and uterus cancer were adjusted for age at diagnosis and cancer spread at diagnosis; all other estimates were adjusted for sex, age at diagnosis, and cancer spread at diagnosis. SHRs are shown on a log scale. Detailed estimates are provided in Table S6.

Supplementary Methods

Data source

We utilized Cancer Institute NSW Enduring Cancer Data Linkage (CanDLe) data, which include NSW Cancer Registry (NSWCR) records for all individuals diagnosed or treated with cancer in NSW between 1972-2021. NSWCR records were the main data source for this study (see details in the following). Within CanDLe, NSWCR data were also linked to:

1) the Cause of Death Unit Record File (COD-URF; Jan-1985 to Dec-2021, used to identify additional non-cancer deaths, see below), and
2) the Admitted Patient Data Collection (APDC; Jan-2001 to Dec-2019; used to identify comorbidities as described below).

Study population and cancer diagnosis records

Individuals with invasive solid cancers were identified from NSWCR records for cancer diagnosis, using ICD-10 codes (C00-C69 and C73-C80; Table S1). This study also analysed the 12 most common cancer types separately: prostate (C61), female breast (C50), melanoma of the skin (C43), colorectal (C18-C20), lung (C33-C34), kidney (C64), pancreas (C25), thyroid (C73), uterus (C54-C55), bladder (C67), liver (C22) and stomach (C16).

Consistent with a previous NSW study that examined the association between SES and risk of cancer death for individuals diagnosed 1980-2008[1], individuals were excluded from all analyses if they met any of the following criteria: diagnosis before 1980 (due to no information on area-based SES), missing exact diagnosis date (i.e., only year known), a first record of brain, central nervous system (CNS) or lympho-haematopoietic cancer (C70-C72;C81-C96) or linkage errors (Figure S1). Records were excluded based on the following criteria: brain/CNS/lympho-haematopoietic cancers, duplicate information, cancer diagnosis solely from death certificate, in-situ cancers, missing values for characteristics (except spread of disease, see statistical analysis section).

We only included cancer diagnoses to the end of 2019, as data for 2020-2021 only covered two years of the 2020-2029 diagnostic decade and trends in 2020-2021 are expected to be influenced by temporary changes during the COVID-19 pandemic.

Death records

NSWCR data were used to determine date of death and cause of death. For date of death, only month and year were available for this analysis; thus, deaths were assigned the 15th date of the relevant month.

For 25,229 individuals (out of 942,241) with no death record in the NSWCR data but a record of non-cancer death in COD-URF, the non-cancer death information from COD-URF was included in the analysis.

ICD-10 codes used to categorise cause of death are shown in Table S1.

Individuals' characteristics

For each individual, we considered the following information from the NSWCR data: area-based SES at diagnosis (included in NSWCR records from the earliest available records in 1980), 10-year period of diagnosis, age at diagnosis, sex, spread of disease at diagnosis, and area-based remoteness of residence at diagnosis.

Area-based socioeconomic status (SES)

Area-based SES was assessed using the census-derived Index of Relative Socio-Economic Disadvantage (IRSD)[2-4], which is composed of 15 indicators that reflect economic and social disadvantage of individuals and households within defined geographical areas at a specific time point. The NSWCR assigns records to quintiles, from 1 (most disadvantaged)' to '5 (least disadvantaged)'[5]. Quintile assignment is based on the diagnosis year and the corresponding census index[5](see NSWCR data dictionary for details).

Spread of disease

Among the 942,241 individuals with invasive solid cancer, 16.4% had unknown spread of disease at diagnosis (ranging from 5% for melanoma cancer to 34% for prostate cancer). Previous studies have suggested that unknown spread of disease in population-based cancer registries is not missing completely at random and the group of cancer cases recorded as unknown spread of disease include individuals with a mixture of stages[6,7]. This means that analyses including unknown spread of disease as a group may be subject to some residual confounding, and complete-case analyses excluding individuals with unknown spread of disease may produce biased results[6,7].

Multiple imputation (MI) has been increasingly used in epidemiological studies[8] and was recommended as the preferred approach for handling missing data[9]. We previously validated the use of MI for prostate cancer using NSWCR data, with the validation enabled by availability of additional information on cancer stage from the NSW Prostate Cancer Care and Outcomes Study (PCOS)[10]. Consistent with previous studies that applied MI to spread of disease for colorectal, melanoma and female breast cancers using simulated data[9,11,12], our validation study suggested that MI for unknown spread of disease data recorded in the NSWCR appears to provide valid estimates in assessing the association between cancer survival and SES[10,13]. In particular, while there was a slight departure from the missing at random assumption when using the "basic" imputation model (including variables available from the NSWCR, as applied in the current study), we found that this "basic" imputation model appeared to still provide relatively unbiased estimates when assessing the association between cancer survival and SES. Notably, the results based on "basic" imputation model and "enhanced" imputation model (including primary treatment information from external PCOS data) were almost identical[10].

Thus, to infer spread of disease where missing in the current study, we applied the "basic" imputation model developed previously by Luo and colleagues[10], imputing "local", "regional" or "distant" spread for those with unknown spread of disease. Logistic regressions were used to select variables for imputation models for all cancers combined and for each specific cancer type. Spread of disease at diagnosis was considered as "unknown" or "known", and variables associated with the odds of "unknown" spread were selected for inclusion in the models: age at diagnosis, sex (if applicable), period of diagnosis, cause of death, vital status and survival time censored at 31/12/2021, and area-level SES. Multinomial logistic regression was performed to impute spread of disease where "unknown", using the Stata package *ice*. Following previous approaches[10,13,14], 35 imputations were used per individual, with this number exceeding the highest percentage of records with

"unknown" spread (i.e., 34% missing for prostate cancer) for the analysis of all solid cancers combined and each of the 12 common cancer types (here, the highest observed percentage was for prostate cancer). Before imputation, 44.2% had local cancer spread, 22.4% regional spread and 17.0% distant spread. After imputation, 53.5% had local spread, 26.2% had regional and 20.4% had distant spread of disease at diagnosis. Descriptive statistics for spread of disease based on NSWCR records and post-MI are shown in Table S3.

Area-based remoteness of residence

Remoteness of residence was assessed using the Accessibility and Remoteness Index of Australia plus. The NSWCR provides remoteness categories as 'major cities', 'inner regional', 'outer regional', 'remote' and 'very remote' from diagnosis 2000 onwards. For diagnoses between 1980 and 1999, we used a similar approach as a previous study on cancer survival by area-based SES[1], mapping 2006 Statistical Local Areas (SLA) to remoteness areas categories[15]. Some SLAs span multiple remoteness categories; thus, data from the Australian Bureau of Statistics[15] include a proportionate assignment of each SLA to one or more remoteness categories (based on the respective proportions of the population).

Thus, for individuals diagnosed 1980-1999, we assigned weights for different remoteness categories based on the SLA at time of diagnosis (sum of weights equal to 1 for each individual). For example, individuals in the SLA "Blue Mountains" were assigned a weight of 0.9246 for 'major cities' and 0.0754 for 'inner regional', and a weight of 0 for 'outer regional' and 'remote/very remote'.

To harmonise the approach across all diagnosis periods, for individuals diagnosed after 2000, the assignment to a single remoteness category was also converted to a weighted assignment (1 for that remoteness category, 0 for all other categories).

For association analyses that adjusted for remoteness of residence, we thus included each remoteness category as a continuous covariate with values ranging from 0 to 1; remote and very remote areas were combined due to smaller populations.

Charlson's Comorbidity Index (CCI)

Charlson's comorbidity Index (CCI) was derived from APDC records using ICD-10-AM codes as specified by Sundararajan et al.[16], with conditions scored as per Quan et al.[17]. Table S2 details the codes and scores for different conditions. For each individual, we considered conditions noted in hospital records up to 2 years prior to the cancer diagnosis. As APDC data were only available from 2001 (and thus do not cover the full relevant periods for diagnosis decades 1980-1989 to 2000-2009), we only calculated the CCI for a sensitivity analysis restricted to individuals diagnosed 2010-2019.

National Comorbidity Index (adapted algorithm)

To assess robustness of results to the choice of comorbidity measure, we derived an adapted version of the National Comorbidity Index (NCI) from APDC ICD-10 diagnoses codes, using published condition lists and weighted scores[18,19](Table S2). As the original NCI uses both hospital and physician billing data and the latter were unavailable in our study, we applied a previously published adapted version that uses hospital records only. As in the CCI analysis, we included conditions captured in the 2 years

prior to cancer diagnosis and restricted calculation to individuals with cancers diagnosed in 2010-2019."

Cancer-specific and overall survival

For the analysis of all solid cancers combined, cancer-specific survival was defined as time (years) from first cancer diagnosis to death from cancer. Individuals were thus included in this analysis based on their first cancer diagnosis. For example, an individual with records for a diagnosis of prostate cancer in 1990 and melanoma in 2000 would be included in the analysis of all solid cancers based on the prostate cancer diagnosis in 1990.

For analysis of a specific cancer type, cancer-specific survival was the time from first diagnosis to death from that specific cancer, with deaths due to other causes, including other cancers, treated as competing events. Individuals with multiple diagnosis records for different cancers were included in each relevant analysis. For example, an individual with records for a diagnosis of prostate cancer in 1990 and melanoma in 2000 would be included in the analyses of specific cancer types for prostate cancer and melanoma accordingly.

Overall survival was defined as the time from first cancer diagnosis to death from any cause.

In the analysis of cancer-specific and overall survival for all solid cancers combined, individuals were included based on their first primary cancer diagnosis only, and those alive at the end of follow-up (15-Dec-2021) were censored.

We estimated 1-, 2- and 5-year crude cancer-specific and overall survival, stratified by 10-year period of diagnosis and cancer spread (post-MI), for all solid cancers combined, and for each cancer. Crude survival was calculated as 1 minus the cumulative incidence function (CIF)[20], using SAS LIFETEST, with estimates combined using Rubin's rule[21] through MIANALYZE. Changes in survival over time were summarized as the absolute percentage difference in 1 and 5-year crude survival between 1980-89 and 2010-19.

As the distribution of cancer types can change over time and impact estimates of crude survival, some studies have suggested constructing weighted survival indices[22-24]. Here, we additionally calculated weighted crude survival estimates as follows. For each of the diagnosis periods 1990-99, 2000-2009, and 2010-2019, we weighted the data for all solid cancers combined to match the cancer type distribution for cancers incidence in NSW in 1980-89. We completed two separate analyses using two different sets of post-stratification weights, based on different aggregation of less common cancer types:

1) separate weights for each of the 12 most common cancer types (as per dedicated survival analyses in the manuscript), and a weight for all remaining cancer types grouped as "other_{12}", and
2) more detailed post-stratification weight, with separate weights for each of the 19 most common cancer types (cancer types comprising ≥0.5% of all diagnoses between 1980-2019), and a weight for the smaller residual category of remaining cancers grouped as "other_{19}".

Associations between SES and risk of death

Associations between SES and risk of death were assessed using competing risks subdistribution hazard regressions, estimating sub-hazard ratios (SHRs) and 95% confidence intervals (CIs). Risk of cancer-specific death was analyzed with R (FASTCRR), and risk of all-cause death with SAS (PHREG).

For the analyses of all-cause deaths, there are no competing events; thus, the competing risks subdistribution hazards model are mathematically equivalent to cause-specific Cox proportional hazards models. This equivalence has been demonstrated in Austin et al.(2016)[20].

For all solid cancers, the analysis was minimally-adjusted for sex, age at diagnosis and cancer type, and stratified by 10-year period of diagnosis. To examine the extent of SES-associated differences in survival that are not solely due to differences in spread of disease at diagnosis, we completed additional analyses that also adjusted for cancer spread (based on spread of disease post-MI, combining estimates from different MI runs by using Rubin's rule). For separate analyses of the 12 most common cancers, we used an analogous approach: minimally-adjusted models for sex (where relevant) and age at diagnosis, stratified by 10-year period of diagnosis: additional analyses also adjusted for cancer spread (post-MI).

We assessed the proportional hazards assumption across SES quintiles in competing risks regression models[25], plotting the transformed variables: log(-log(1-CIF)) against log(time) for the CIF and time to cancer death, respectively.

To verify trends over time, we completed further analyses that explicitly tested for interactions between diagnosis year (as a continuous variable) and SES, using Wald's chi-squared tests. These analyses were not stratified by diagnosis period, combining the full data for 1980-2019. Specifically, we conducted analyses of cancer-specific deaths for:

1) All solid cancers combined: model including cancer type, age, sex, diagnosis year, SES, interaction between diagnosis year and SES;
2) All solid cancers combined: model including cancer type, age, sex, cancer spread (post-MI), diagnosis year, SES, interaction between diagnosis year and SES (with estimates combined from different MI runs using Rubin's rule);
3) Each of the 12 most common cancers: models including age, sex (where relevant) and cancer spread (post-MI), diagnosis year, SES, and the interaction between diagnosis year and SES (with estimates combined from different MI runs using Rubin's rule),

Sensitivity analyses

We conducted multiple sensitivity analyses to examine the impact of different factors on the association between SES and cancer survival, as described in detail below. All sensitivity analyses were based on the main analysis adjusting for sex, age at diagnosis, cancer type, and spread of disease at diagnosis.

For the analysis of all solid cancers combined, cancer-specific and all-cause deaths:

1) To examine the robustness of associations between SES and cancer survival when using spread of disease post-MI versus pre-MI: instead of spread of disease post-MI, adjusting for spread of disease as per NSWCR record (i.e. include "unknown" as a separate category).
2) To examine the extent of SES-associated differences in survival after adjustment for comorbidities: Adjusting for CCI in addition to sex, age at diagnosis, cancer type, and spread of disease at diagnosis (restricted to 2010-2019 diagnosis period due to limited availability of CCI data).
3) To examine the extent of SES-associated differences in survival after adjustment for remoteness of residence: Adjusting for area-based remoteness of residence in addition to sex, age at diagnosis, cancer type, and spread of disease at diagnosis.

4) To examine the extent of SES-associated differences in survival after adjustment for both comorbidities and remoteness of residence: Adjusting for CCI and area-based remoteness of residence, in addition to sex, age at diagnosis, cancer type, and spread of disease at diagnosis (restricted to 2010-2019 diagnosis period due to limited availability of CCI data).
5) To examine the robustness of associations between SES and cancer survival to different censoring of people with multiple cancer diagnoses: For individuals with more than one cancer diagnosis, censoring individuals at the second occurrence of any cancer.

For the analysis of all solid cancers combined and cancer-specific death:

6) To examine the extent of SES-associated differences in survival after adjustment for comorbidities, using an alternative comorbidity index: Adjusting for NCI in addition to sex, age at diagnosis, cancer type, and spread of disease at diagnosis (restricted to 2010-2019 diagnosis period due to limited availability of NCI data).

As the association between SES and risk of cancer-specific death was particularly large for prostate and breast cancers, two of the most common cancers, we also completed sensitivity analyses to examine the extent of association between SES and cancer survival for all solid cancers combined except prostate and breast:

7) Excluding individuals with a first primary invasive cancer of prostate or breast cancer;
8) Excluding individuals with a first primary invasive cancer of prostate or breast cancer and adjusting for comorbidities (CCI) and area-based remoteness of residence, in addition to sex, age at diagnosis, cancer type, and spread of disease at diagnosis (restricted to 2010-2019 diagnosis period due to limited availability of CCI data).

Analogous to the weighted analysis of crude survival estimates, we completed sensitivity analyses of associations between cancer-specific deaths and SES for all solid cancers combined, weighting data by cancer type. Here, we completed two analyses using the same two sets of weights as described above for estimates of crude survival, to match the cancer type distribution in each decade to diagnosis data in 1980-1989. In each weighted dataset, we included adjustments for sex, age at diagnosis, cancer type (12 common cancers and group of remaining other cancers), and spread of disease at diagnosis. We also completed sensitivity analyses for dedicated analyses of specific cancer types:

1) For each of prostate cancer, breast cancer, and melanoma (separate analyses), cancer-specific deaths: excluding individuals with localised disease at diagnosis (to examine the potential impact of overdiagnosis);
2) For breast, colorectal, and prostate cancers (separate analyses), cancer-specific and all-cause deaths: defining diagnosis periods based on key changes to screening programs or early detection tests (rather than decades) to capture potential resulting changes in cancer patterns, and ensure the robustness of associations between SES and cancer survival.
    a. Prostate cancer: diagnosis periods of 1980-89,1990-99, 2000-09, 2010-14, 2015-19, aligning with introduction of PSA testing in the Medicare Benefits Schedule in 1989, and changes to PSA testing guidelines from 2014[26,27]).
    b. Breast cancer: diagnosis periods of 1980-91, 1992-99, 2000-08, 2009-13, 2014-19, aligning with the implementation of the national screening program in 1991, transition to digital mammography in 2008, and extension of the national screening program to 70-74 years age group from 2013 .

    c. Colorectal cancer: diagnosis periods of 2000-06 and 2007-19, based on the introduction of the national bowel cancer screening program in 2006[26]).
3) For cancers with small sample size for some age groups leading to wide 95%CIs for the SHRs for that age group (and thus potentially less stable results overall), combine age groups into broader categories to ensure the robustness of associations between SES and cancer survival:
    a. Prostate cancer: combine age groups 0-39 and 40-49 years into 0-49 years;
    b. Thyroid cancer: combine multiple age groups to categorise age as 0-59 and ≥60 years.

For all sensitivity analyses, we compared the SHR estimates for SES to the results of the relevant main analysis, considering the absolute difference in SHRs, the direction of the associations, as well as changes in significance (i.e., 95% CIs include unity).

Supplementary Results and Discussion

Crude cancer-specific and overall survival

From 1980-1989 to 2010-2019, cancer-specific survival increased for all solid cancers combined and for 11 out of 12 common cancers (except bladder). For all solid cancers combined, crude 1-year cancer specific survival improved from 69.5% in 1980-89 to 85.3% in 2010-19 (Figure S2a, Table S4). The largest improvements (≥20% absolute change) in 1-year cancer-specific survival from 1980-89 to 2010-19 were for lung, kidney, liver and stomach cancers (Figure S2c). For bladder cancer, survival declined pre-2000 then stabilized. The decline has been previously attributed to changes in tumor coding and increasing age at diagnosis[28-30], consistent with national estimates[29].

Crude survival estimates weighted to cancer type distribution in 1980-1989 were highly similar to estimates in the main analysis (absolute differences in the increase in cancer survival between 1980-89 and 2010-2019 was <1% for each of 1-, 2- and 5-year survival).

Sensitivity analyses and discussion: all solid cancers combined

For all solid cancers combined, sensitivity analyses generally yielded similar patterns of association between SES and risk of cancer death to the main analyses adjusting for sex, age, cancer type and cancer spread. The full details of all sensitivity analyses are provided in Table S7.

In particular, excluding prostate and breast cancers only slightly reduced the SHR for most versus least disadvantaged areas in the 2010-2019 diagnosis period, from SHR=1.35 (95%CI:1.32-1.38) to SHR=1.31 (95%CI:1.28-1.34). This suggests the significant variation in survival by SES for all solid cancers combined was not due to these two common cancers only.

Moreover, association estimates from weighted analyses (matching cancer type distribution in each decade to 1980-89) yielded SHR estimates highly similar to the main analysis (absolute difference ≤0.02).

Considering analyses of all solid cancer combined with adjustment for comorbidities, an adjustment for CCI in addition to sex, age at diagnosis, cancer type, and spread of disease at diagnosis yielded similar SHR estimates to the main analysis, with only a slight attenuation in association (Table S7). Notably, adjustment for NCI (an alternative comorbidity index) yielded similar results to adjustment for CCI (Table S7). This result is well-aligned with previous Australian cancer studies reporting similar mortality prediction using CCI and NCI indices[31,32]. While SHR estimates from different models are not directly comparable, given the similarity in results and prior studies validating the CCI in the Australian cancer context[16,17], the CCI was used for further analyses including adjustment for comorbidities (see below).

Among all sensitivity analyses for all solid cancers combined, the largest reduction in SHRs compared to the main analysis was observed in the sensitivity analysis that excluded prostate and breast cancers and also adjusted for CCI and remoteness, with the SHR for most versus least disadvantaged areas in the 2010-2019 diagnosis period reduced (by 0.09) to SHR=1.27 (95%CI:1.23-1.30), and the association remaining significant.

This indicates that CCI and remoteness of residence (as well as prostate/breast cancers) explain some of the variation in cancer survival by SES. However, we note that CCI, as ascertained in our study,

provided incomplete information on individuals' comorbidities and overall health. For example, key differences in health status not captured in hospital diagnosis codes that could impact treatment options and preferences include performance status and psychological health [33].

Main and sensitivity analyses for each of the 12 most common cancers

For 10 of the 12 common cancers, there was some evidence of an association between risk of cancer death and area-based disadvantage for those diagnosed in 2010-19 (i.e., 95% CIs for at least two SHR estimates did not include unity), including prostate, breast, melanoma, colorectal, lung, pancreatic, uterine, bladder, liver and stomach cancers. For eight of these cancers (except uterine and liver cancer), there was a trend towards widening disparities by SES between 2000-09 and 2010-19 (i.e. higher SHR estimates in 2010-19 than in 2000-09, Figure 2, Table S5). For all eight cancers, the additional analyses showed significant interactions between SES and year of diagnosis ($p<0.001$), supporting variability in effects across time.

For seven of the cancers (prostate, breast, melanoma, colorectal, lung, bladder, and stomach cancers), we found an indication that risk of cancer death for most versus least disadvantaged areas may have increased from 2000-09 to 2010-19 (SHR estimate for 2010-19 outside 95%CIs for 2000-09), though we note that (here and below) this comparison is limited by SHR estimates from period-stratified analyses not being directly comparable between periods.

For each of the 12 cancers, detailed results are described below.

Prostate cancer

Risk of death from prostate cancer increased with area-based disadvantage from 1990 onwards, coinciding with the introduction of PSA testing in 1989 (model adjusting for age and cancer spread; Table S5). Disparities widened over time, with the SHR for the most versus least disadvantaged areas in 1990-99 increasing from 1.08 (95%CI:1.01-1.15) to 1.29 (95%CI:1.20-1.39) in 2000-09 and 1.58 (95%CI:1.44-1.74) in 2010-19. By 2010-19, risk of death from prostate cancer was significantly higher for all four quintiles of area-based disadvantage compared to the least disadvantaged areas. Associations for all quintiles showed increases across time, e.g., quintile 4 versus 5 (least disadvantaged) in 1990-99 SHR=1.05 (95%CI:0.96-1.15), in 2000-09 SHR=1.08 (95%CI:0.99-1.17) and in 2010-19, SHR=1.22 (95%CI:1.10-1.37).

Excluding individuals with localized disease at diagnosis attenuated many associations between cancer-specific deaths and area-based disadvantage (absolute reduction in SHRs up to 0.17), with only one of five associations for 1980-2009 remaining significant (Table S7). For example, for 2000-09, the association between cancer-specific death and most disadvantaged areas (versus least disadvantaged areas) changed from SHR=1.29 (95%CI:1.20-1.39) including localized disease to SHR=1.12 (95%CI:1.00-1.26) excluding localized disease. However, for diagnoses in 2010-19, the exclusion of localized disease had little impact on SHRs, and risk of cancer-specific death remained significantly elevated for all area-based SES quintiles (versus the least disadvantaged quintile).

Defining diagnoses periods based on key changes in PSA testing guidelines did not yield substantial changes in results, with wide confidence intervals including estimates for similar diagnoses periods in the main analysis. For example, we found associations for the most versus least disadvantaged areas of SHR=1.51 (95%CI:1.32-1.72) in 2010-14 and SHR=1.72 (95%CI:1.08-2.72) in 2015-19, with

confidence intervals including the estimate of SHR=1.58 (95%CI:1.44-1.74) in 2010-19 (all adjusting for age and cancer spread).

Breast cancer

The risk of death from breast cancer increased with higher socioeconomic disadvantage across all periods of diagnoses (model adjusting for age and cancer spread; Table S5). In 2010-19, risk of death from breast cancer was significantly higher for all four quintiles of area-based disadvantage compared to the least disadvantaged areas (Figure 2, Table S5). The associations ranged from SHR=1.11 (95%CI:1.02-1.22) for area-based SES quintile 4 versus 5 (least disadvantaged), to SHR=1.35 (95%CI:1.23-1.48) for area-based SES quintile 1 (most disadvantaged) versus 5 (least disadvantaged). Notably, differences in risk of breast cancer death between the most and least disadvantaged areas widened between 2000-09 and 2010-19 (SHR estimate for 2010-19 not within 95% CIs for 2000-09).

Excluding individuals with localized disease at diagnosis resulted in minimal changes to SHRs. For example, the SHR for the most versus least disadvantaged areas in 2010-19 was SHR=1.35 (95%CI:1.23-1.48) when including localized disease, with a similar result of SHR=1.31 (95%CI:1.18-1.45) when excluding localized disease.

Similarly, defining diagnosis periods based on the introduction and significant changes to the national breast screening programme did not yield a substantial change in results. For risk of breast cancer death, the SHR for the most versus least disadvantaged areas was SHR=1.27 (95%CI:1.14-1.42) in 2009-13 and SHR=1.44 (95%CI:1.26-1.63) in 2014-19, with confidence intervals including the estimate of SHR=1.35 (95%CI:1.23-1.48) in 2010-19.

Melanoma

Across all periods of diagnosis, risk of melanoma death after diagnosis was higher for individuals in the most disadvantaged areas compared to the least disadvantaged areas (model adjusting for age, sex and cancer spread; Table S5). Results for other quintiles of area-based disadvantage were more variable across time. Notably, in 2010-19, risk of death from melanoma was significantly higher for all four quintiles of area-based disadvantage compared to the least disadvantaged, with e.g. quintile 3 versus 5 (least disadvantaged) SHR=1.35 (95%CI:1.18-1.54). For the most versus least disadvantaged areas, the SHR increased from 1.18 (95%CI:1.07-1.30) in 2000-09 to 1.49 (95%CI:1.32-1.68) in 2010-19.

Excluding individuals with localized disease at diagnosis attenuated the associations between risk of death from melanoma and area-based disadvantage (absolute reduction in SHRs up to 0.16), with few associations remaining significant for 1980-2009 (Table S7). For example, the risk of melanoma death for those living in the most versus least disadvantaged areas in 2000-09 changed from SHR=1.18 (95%CI:1.07-1.30) including localized disease changed to SHR=1.15 (95%CI:1.00-1.33) excluding localized disease. However, the results for 2010-19 were largely unaffected by this exclusion, with similar point estimates and only slightly wider 95%CIs, e.g. most versus least disadvantaged areas SHR=1.49 (95%CI:1.32-1.68) including localized disease and SHR=1.51 (95%CI:1.29-1.78) excluding localized disease. Notably, risk of melanoma-specific death remained significantly elevated for all four categories of area-based disadvantage (versus least disadvantaged areas) in 2010-19 even after exclusion of localized disease.

Colorectal cancer

Across all diagnosis periods, risk of death from colorectal cancer was consistently higher in areas with greater disadvantage (significant associations for area-based SES quintiles 1 (most disadvantaged) and 2 versus 5 (least disadvantaged); model adjusting for age, sex and cancer spread; Table S5). Notably, for the most disadvantaged areas compared to the least disadvantaged areas, the association with risk of death from colorectal cancer increased from SHR=1.18 (95%CI:1.11-1.25) in 2000-09 to SHR=1.27 (95%CI:1.20-1.34) in 2010-19.

Sensitivity analyses that considered diagnosis periods of 2000-06 and 2007-19 instead of 2000-09 and 2010-19 did not yield any appreciable change in results (Table S7).

Lung cancer

Across all diagnosis periods, risk of death from lung cancer was consistently higher in the most disadvantaged areas (versus least disadvantaged areas; model adjusting for age, sex and cancer spread; Table S5). Associations with other quintiles of area-based disadvantage were also significant from 1990 onwards. Overall, disparities in lung cancer death remained relatively stable between 1980 and 2009, but increased slightly in 2010-19. In particular, for the most versus least disadvantaged areas, the SHR increased from SHR=1.18 (95%CI:1.12-1.23) in 2000-09 to SHR=1.26 (95%CI:1.20-1.32) in 2010-19.

Kidney cancer

There was only very slight evidence for an association between risk of death from kidney cancer and area-based disadvantage for diagnoses in 2010-19 (area-based SES quintile 2 versus 5 (least disadvantaged) SHR=1.20; 95%CI:1.00-1.44; model adjusting for age, sex and cancer-spread; Table S5), with association for most versus least disadvantaged areas not reaching statistical significance (SHR=1.18; 95%CI:0.99-1.41). There were no significant associations in 1990-99 and 2000-09.

Pancreatic cancer

Risk of death from pancreatic cancer was significantly higher for all four quintiles of area-based disadvantage compared to the least disadvantaged areas from 2000 onwards (model adjusting for age, sex and cancer spread; Table S5). Results were largely similar for diagnoses in 2000-09 and 2010-19, e.g. most versus least disadvantaged areas SHR=1.23 (95%CI:1.13-1.34) in 2000-09 and SHR=1.26 (95%CI:1.18-1.36) in 2010-19.

Thyroid cancer

There was no clear association between SES and risk of death from thyroid cancer, with wide variability in SHR estimates within and between periods of diagnosis, and wide 95% CIs (model adjusting for age, sex, and cancer spread; Table S5).

A sensitivity analysis combining age groups into categories of 0-59 and ≥60 years did not change the results (Table S7).

Uterine cancer

There was some evidence for the association between SES and death from uterine cancer among those diagnosed from 2000 onwards (model adjusting for age and cancer spread; Table S5). In 2000-09, for the most versus least disadvantaged areas, the SHR was 1.25 (95%CI:1.00-1.55) and in 2010-19 the SHR=1.25 (95%CI:1.05-1.48). The SHRs between 2000-09 and 2010-19 varied and there was no observed widening of SES disparities.

Bladder cancer

For diagnoses up to 1999, there was no clear association between SES and death from bladder cancer. For diagnoses in 2000-09, there was a slightly higher risk of bladder cancer death for the most versus least disadvantaged area, with SHR=1.14 (95%CI:1.01-1.29) (model adjusting for age, sex and cancer spread; Table S5). Associations between SES and cancer-specific death strengthened for diagnoses 2010-19, including a significant association for the most versus least disadvantaged areas, SHR=1.39 (95% CI:1.23-1.57), and analogous increases in risk for SES quintiles 2 and 3 versus 5 (least disadvantaged). There was evidence that the gap in risk of bladder cancer death by SES was widening between 2000-09 and 2010-19; for example, the SHR estimate for most versus least disadvantaged areas based on diagnoses in 2010-19 was outside the 95% CIs for the estimates based on diagnoses in 2000-09.

Liver cancer

Risk of death from liver cancer was significantly higher in most disadvantaged areas (versus least disadvantaged areas) from 1990 onwards (model adjusting for age, sex and cancer spread; Table S5). While there were wide and overlapping 95% CIs for estimates, we note there was a slight trend towards reduced point estimates over time, e.g. for most vs least disadvantaged areas, SHR=1.31 (95%CI:1.11-1.54) in 1999-99, SHR=1.24 (95%CI:1.10-1.41) in 2000-09 and SHR=1.14 (95%CI:1.04-1.25) in 2010-19.

Stomach cancer

In 2010-19, risk of death from stomach cancer increased with area-based disadvantage (model adjusting for age, sex and cancer spread; Table S5). For the most versus least disadvantaged areas in that period, we estimated SHR=1.28 (95%CI:1.16-1.42); the risk of death was also significantly higher for SES quintile 2 versus 5 (least disadvantage) in the same period (SHR=1.17, 95%CI:1.05-1.32). There was evidence that the gap in SES disparity widened between 2000-09 and 2010-19; for example, the SHR estimate for most versus least disadvantaged areas based on diagnoses in 2010-19 was outside the 95% CIs for the estimates based on diagnoses in 2000-09 (SHR=1.09, 95%CI:0.98-1.21).

Proportional hazards assumption

Visual assessments of the log-log plots for all solid cancers combined and the 12 most common cancer types indicated that the proportional hazards assumption was generally met.

Use of multiple imputation method for unknown spread of disease

The main strength of using MI for unknown spread of disease is that where application of MI is valid, it allows for a more complete examination of the association between SES and cancer survival, with statistical control for the effects of spread of disease at diagnosis (compared to analyses that include unknown spread of disease as a group). In addition, using MI for unknown spread of disease accounts for the uncertainty due to missing data[13,34]. The use of MI for unknown spread of disease for prostate cancer in NSWCR data has been validated previously using real-world data[10]. We note it is an inherent challenge of validating MI approach that the missing data are unknown[10] and a more general validation across all cancers was not possible.

The results of MI could be affected if there are major departures from the assumption of data being missing at random; this could also affect the results of subsequent analyses. As per the above, our previous validation study for prostate cancer suggests that the "basic" imputation model (as applied in the current study) appears to provide relatively unbiased estimates when assessing the association between cancer survival and SES, supporting the use of MI in the current study.

Notably, in the current study, spread of disease was a covariate and the association results for SES and cancer survival were highly consistent between analyses that included spread of disease post-MI or spread of disease data as recorded in the registry (including a separate category of "unknown" spread of disease; see Table S7). This supports the robustness of results for association between cancer survival and SES as described in this study.

References

1. Tervonen HE, Aranda S, Roder D, *et al*. Cancer survival disparities worsening by socio-economic disadvantage over the last 3 decades in New South Wales, Australia. BMC Public Health 2017;17(1):691.
2. Australian Bureau of Statistics. *Census of Population and Housing Socio-Economic Indexes for Areas 2011 Quality Declaration*. https://www.abs.gov.au/Ausstats/abs@.nsf/0/BEC86C4146B4A10CCA258259000BA7F1?OpenDocument. Accessed Nov 15, 2023.
3. Australian Bureau of Statistics. *SEIFA Using and interpreting SEIFA*. https://www.abs.gov.au/websitedbs/censushome.nsf/home/seifahelpansuis?opendocument&navpos=260. Accessed Nov 15, 2023.
4. Australian Bureau of Statistics. *Census of Population and Housing Socio-Economic Indexes for Australia IRSD*. https://www.abs.gov.au/ausstats/abs@.nsf/lookup/2033.0.55.001main+features100052011#:~:text=The%20Index%20of%20Relative%20Socio,only%20measures%20of%20relative%20disadvantage. Accessed Nov 15, 2023.
5. NSW Centre for Health Record Linkage. *CHeReL master linkage key NSW Cancer Registry data dictionary. Date accessed 15 Nov 2023*. https://www.cherel.org.au/datasets.
6. Gurney J, Sarfati D, Stanley J, *et al*. Unstaged cancer in a population-based registry: prevalence, predictors and patient prognosis. Cancer Epidemiol 2013;37(4):498-504.
7. Luo Q, Yu XQ, Cooke-Yarborough C, *et al*. Characteristics of cases with unknown stage prostate cancer in a population-based cancer registry. Cancer Epidemiol 2013;37(6):813-9.
8. Hayati Rezvan P, Lee KJ, Simpson JA. The rise of multiple imputation: a review of the reporting and implementation of the method in medical research. BMC Med Res Methodol 2015;15:30.
9. Marshall A, Altman DG, Royston P, *et al*. Comparison of techniques for handling missing covariate data within prognostic modelling studies: a simulation study. BMC Med Res Methodol 2010;10:7.
10. Luo Q, Egger S, Yu XQ, *et al*. Validity of using multiple imputation for "unknown" stage at diagnosis in population-based cancer registry data. PLoS One 2017;12(6):e0180033.
11. Eisemann N, Waldmann A, Katalinic A. Imputation of missing values of tumour stage in population-based cancer registration. BMC Med Res Methodol 2011;11:129.
12. Falcaro M, Nur U, Rachet B, *et al*. Estimating excess hazard ratios and net survival when covariate data are missing: strategies for multiple imputation. Epidemiology 2015;26(3):421-8.
13. White IR, Royston P, Wood AM. Multiple imputation using chained equations: Issues and guidance for practice. Stat Med 2011;30(4):377-99.
14. Graham JW, Olchowski AE, Gilreath TD. How many imputations are really needed? Some practical clarifications of multiple imputation theory. Prev Sci 2007;8(3):206-13.
15. Australian Bureau of Statistics. *1216.0.15.003 Australian Standard Geographical Classification (ASGC) Remoteness Area Correspondences, 2006*. https://www.abs.gov.au/AUSSTATS/abs@.nsf/DetailsPage/1216.0.15.0032006?OpenDocument.
16. Sundararajan V, Henderson T, Perry C, *et al*. New ICD-10 version of the Charlson comorbidity index predicted in-hospital mortality. J Clin Epidemiol 2004;57(12):1288-94.
17. Quan H, Li B, Couris CM, *et al*. Updating and Validating the Charlson Comorbidity Index and Score for Risk Adjustment in Hospital Discharge Abstracts Using Data From 6 Countries American Journal of Epidemiology 2011;173(6):676-682.
18. Klabunde CN, Potosky AL, Legler JM, *et al*. Development of a comorbidity index using physician claims data. J Clin Epidemiol 2000;53(12):1258-67.
19. National Cancer Institute. *Table describing changes in codes for NCI Comorbidity Index*. https://healthcaredelivery.cancer.gov/seermedicare/considerations/comorbidity-table.html. Accessed Aug 1, 2024.

20. Austin PC, Lee DS, Fine JP. Introduction to the Analysis of Survival Data in the Presence of Competing Risks. Circulation 2016;133(6):601-9.
21. UCLA: Statistical Consulting Group. *Multiple Imputation in SAS Part 1*. . https://stats.oarc.ucla.edu/sas/seminars/multiple-imputation-in-sas/mi_new_1/.
22. Johnson CJ, Weir HK, Mariotto A*, et al*. Construction of a North American Cancer Survival Index to Measure Progress of Cancer Control Efforts. Prev Chronic Dis 2017;14:E81.
23. Morawski BM, Weir HK, Johnson CJ. Five-Year U.S. Trends in the North American Cancer Survival Index, 2005–2014. American Journal of Preventive Medicine 2020;58(3):453-456.
24. Quaresma M, Coleman MP, Rachet B. 40-year trends in an index of survival for all cancers combined and survival adjusted for age and sex for each cancer in England and Wales, 1971-2011: a population-based study. Lancet 2015;385(9974):1206-18.
25. Pintilie M. Modelling in the Presence of Competing Risks. In. *Competing Risks: A Practical Perspective*: John Wiley & Sons, Ltd; 2006, 87-114.
26. Olver I, Roder D. History, development and future of cancer screening in Australia. Public Health Research & Practice 2017;27(3 DOI - http://dx.doi.org/https://doi.org/10.17061/phrp2731725):e2731725.
27. Pathirana T, Sequeira R, Del Mar C*, et al*. Trends in Prostate Specific Antigen (PSA) testing and prostate cancer incidence and mortality in Australia: A critical analysis. Cancer Epidemiology 2022;77:102093.
28. Australian Institute of Health and Welfare. Cancer survival and prevalence in Australia: period estimates from 1982 to 2010. Asia Pac J Clin Oncol 2013;9(1):29-39.
29. Australian Institute of Health and Welfare. Cancer in Australia 2021. Cancer series no. 133. Cat. no. CAN 144. Canberra: AIHW. In; 2021.
30. Tempo JA, Sii S, Ischia J*, et al*. Lessons from a population-based bladder cancer registry: exploring why survival is not improving. BJU International 2024;133(6):699-708.
31. Pule L, Buckley E, Niyonsenga T*, et al*. Optimizing the measurement of comorbidity for a South Australian colorectal cancer population using administrative data. Journal of Evaluation in Clinical Practice 2020;26(4):1250-1258.
32. Tiruye T, Roder D, FitzGerald LM*, et al*. Prognostic value of comorbidity measures among Australian men with non-metastatic prostate cancer. Cancer Epidemiology 2023;87:102482.
33. Afshar N, English DR, Milne RL. Factors Explaining Socio-Economic Inequalities in Cancer Survival: A Systematic Review. Cancer Control 2021;28:10732748211011956.
34. Little RJ, Rubin DB. *Statistical Analysis with Missing Data*: John Wiley & Sons; 2002.
